# Supplementary material for: A Critical View of the Application of the APEX Software (Aqueous Photochemistry of Environmentally-Occurring Xenobiotics) to Predict Photoreaction Kinetics in Surface Freshwaters
Source: Molecules. 2019 Dec 18;25(1):9. doi: 10.3390/molecules25010009 (PMC7017383; doi:10.3390/molecules25010009)
Supplement: Supplementary file 1 [file molecules-25-00009-s001.zip › 3.2.4_gcc-4.4.0.zip!/3.2.4_gcc-4.4.0/3.2.4_gcc-4.4.0/doc/octave/html/liboctave/Nonlinear-Equations.html]

Nonlinear Equations - Octave C++ Classes


Next: Optimization,
Previous: Nonlinear Functions,
Up: Top


---

## 8 Nonlinear Equations

— : **NLEqn\_options** (void)  
— : **NLEqn\_options** (const NLEqn\_options &opt)

— : NLEqn\_options& **operator =** (const NLEqn\_options &opt)

— : void **init** (void)

— : void **copy** (const NLEqn\_options &opt)

— : void **set\_default\_options** (void)

— : void **set\_tolerance** (double val)

— : double **tolerance** (void)

— : **NLEqn** (void)  
— : **NLEqn** (const ColumnVector&, const NLFunc)  
— : **NLEqn** (const NLEqn &a)

— : NLEqn& **operator =** (const NLEqn &a)

— : void **resize** (int n)

— : void **set\_states** (const ColumnVector &x)

— : ColumnVector **states** (void) const

— : int **size** (void) const

— : ColumnVector **solve** (void)  
— : ColumnVector **solve** (const ColumnVector &x)

— : ColumnVector **solve** (int &info)  
— : ColumnVector **solve** (const ColumnVector &x, int &info)
